# Sickle cell disease, malaria and dengue fever: a case of triple jeopardy

**DOI:** 10.1093/jtm/taz070

**Published:** 2019-09-12

**Authors:** Per O Iversen, Mclean Abisay, Felister Seleki, Mtebe Majigo, Lucio Luzzatto, Julie Makani

**Affiliations:** 1 Department of Haematology and Blood Transfusion, Muhimbili University of Health and Allied Sciences, Dar es Salaam, Tanzania; 2 Department of Haematology, Muhimbili National Hospital, Dar es Salaam, Tanzania; 3 Department of Microbiology and Immunology, Muhimbili University of Health and Allied Sciences, Dar es Salaam, Tanzania

Tanzania has one of the world’s largest populations of patients with sickle cell disease (SCD), a major inherited hemoglobinopathy characterized by anemia and many potential complications, in particular painful vaso-occlusive crisis and infections. The two tropical infectious diseases malaria and dengue fever are also prevalent in Tanzania, and their signs and symptoms overlap with those of SCD. Whereas both concurrent SCD and malaria, SCD and dengue and malaria and dengue have been described,^1–3^ there is apparently no report of simultaneous malaria and dengue in SCD. Here, we present the first report of a patient with this triple-disease manifestation.

An otherwise healthy 34-year-old Tanzanian woman with known SCD was admitted to the Muhimbili National Hospital in Dar es Salaam following 1 week of symptoms of anemia, fever and pain, the latter resembling her previous episodes of vaso-occlusive crisis. The patient fell sick during one of the largest outbreaks of dengue fever in Dar es Salaam, with about 1200 confirmed cases the first 5 months of 2019 according to the Ministry of Health (https://reliefweb.int/report/united-republic-tanzania/tanzania-fights-outbreak-dengue-hits-three-regions). She was diagnosed with homozygous SCD (HbSS) 9 years before and had been admitted to hospital five times for complications of SCD (vaso-occlusive crises and a knee abscess). Her only medication was folic acid (5 g/day). In-hospital routine examination revealed that she had been infected with both *Plasmodium falciparum* malaria (diagnosed by microscopic examination of the parasite on freshly prepared blood slides) and dengue virus. We diagnosed the dengue infection by the combined presence of the dengue NS1 antigen and anti-dengue IgM antibody on hospital admission. At outpatient control 3 months after discharge from hospital, the NS1 antigen was no longer detectable and anti-dengue IgG antibody was positive. Although determination of the dengue serotype for our patient was not available, the circulating serotype for the current dengue outbreak was serotype 1. Tuberculosis was not detected.

The patient was managed with intravenous fluids and anti-pain medication in addition to a 1-day course of intravenous artesunate followed by 3 days of oral therapy with artemether/lumefantrine as treatment of uncomplicated malaria. Antibiotics were not given, and there was no indication for blood or platelet transfusion. Her clinical condition improved markedly upon treatment, and she was discharged from hospital after 10 days. Subsequent outpatient controls were uneventful. Laboratory values are summarized in Table 1.

**Table 1:** Laboratory findings during and after hospitalization of the SCD patient^a^

**Laboratory findings**	**Day 1 (admission)**	**Day 3**	**Day 5**	**Day 10 (discharge)**	**Day 90 (outpatient)**
*P falciparum* parasite count per 500 WBC	138	-	-	0	-
Dengue NS1 antigen present (yes/no)	Yes	-	-	-	No
Dengue antibody-type	IgM	-	-	-	IgG
WBC (reference 4–10 × 10^9^/l)	12.6	12.8	11.0	11.4	10.2
Hb (reference 12–15 g/dl)	7.6	7.4	7.3	7.7	8.6
Platelets (reference 150–410 × 10^9^/l)	220	201	192	228	399

^a^Except for Hb (steady-state 7.5 to 8.5 g/dl), no other pre-admission laboratory values were available as the patient was referred from a low-sourced health center.

WBC—white blood cells; Hb—hemoglobin; Ig—immunoglobulin

Notably, the malaria and dengue infections together did not affect Hb appreciably, possible due to prompt malaria treatment and non-severe form of dengue. Moreover, whereas malaria and dengue usually lead to thrombocytopenia and leukopenia, increased blood levels of leukocytes and platelets are frequent in SCD, possibly explaining the near normal leukocyte and platelet counts in our patient.

Central to the pathogenesis of SCD is the expression of sickle Hb in the erythrocytes, and these cells are also the primary target of the intracellular cycle of *Plasmodium falciparum*. Intriguingly, the erythrocytes are possibly targeted also by dengue virus because they can be opsonized by immune complexes formed in the course of dengue infection.^5^ Furthermore, SCD patients show signs of altered function of the vascular endothelium, and disruption of this barrier is also evident in malaria and dengue infections, possibly mediated at least in part by oxidative stress in all three diseases.^4^^,^^5^ Interestingly, while heterozygous SCD seems to be protected against severe malaria, SCD patients appear to be at a higher risk than homozygous SCD of contracting severe dengue.^1^^,^^2^

SCD, malaria and dengue fever are common and serious disorders in Africa. Although malaria can be treated successfully, few SCD patients receive symptomatic, disease-modifying or curative treatment and there is no specific treatment for dengue. Similarity in their clinical manifestations may cause delay in implementing appropriate treatment. Vigilance is therefore required in areas endemic of these three disorders.

## Author Contributions

P.O.I. treated the patient and drafted the manuscript. E.A. and F.S. diagnosed and treated the patient. M.M. contributed with dengue diagnostics. L.L. and J.M. provided inputs to content and writing of the paper. All authors read and approved the final version of the manuscript.
